# Picric Acid as a Test for Albumen and Sugar in the Urine

**Published:** 1883-08

**Authors:** George Johnson


ON PICRIC ACID AS A TEST FOR ALBUMEN AND SUGAR IN THE URINE. BY GEORGE JOHNSON, M.D., F.R.S.—For the detection of albumen, Dr. Johnson recommends that this acid should "be used in the form of a saturated aqueous solution, or in the form of powder or crystals. The aqueous solution is most suitable for home use, while the powder or crystals may conveniently be carried in a urinary pocket test-case. A saturated aqueous solution may be quickly made by adding about fifty times the bulk of boiling distilled or rain water to the powder or crystals; a portion of the acid will crystallize out on cooling, leaving a transparent yellow supernatant liquid. This solution being added to an equal volume of albuminous urine in a test-tube, immediately coagulates the albumen. The coagulated picrate of albumen is soluble in alkalies; if, therefore, the urine be highly alkaline, it must be acidulated by a vegetable or a mineral acid before adding the picric acid solution. In my numerous testings for albumen with picric acid, I have not once found it necessary to acidulate the urine. The picric acid solution is itself sufficiently acid to dissolve the phosphatic sediment which results from boiling a neutral or alkaline specimen of urine. To detect a very minute quantity of albumen, the following method is the best: Into a test-tube about six inches long, the urine is poured to within two inches of the top; then, the tube being held in a slanting position, about an inch of the picric acid solution is gently poured on the surface of the urine, where, in consequence of its low specific gravity (1003), it only partly mixes with the upper layer of the urine; and, as far as the yellow color of the picric solution extends, there will be more or less turbidity from coagulated albumen, contrasting with the pellucid sustained urine below. If, then, the tube be placed in a stand, the coagulated albumen will gradually subside, and form a delicate horizontal film at the junction of the colored and the unstained stratum of urine, the yellow liquid above and the uncolored urine below being quite free from turbidity. If the urine should be turbid with urates, it must be cleared by heat before the addition of the picric acid solution.

"As a result of numerous observations, I have arrived at the conclusion that the picric acid supplied in this way is a more delicate, and therefore more trustworthy, test for albumen than nitric acid in cold urine, whether the latter be employed by the method of dropping the acid into the cold urine, or by pouring the urine on the acid previously placed in the tube. The simplest and most satisfactory mode of comparing the two tests as regards their relative delicacy, is to dilute a specimen of albumi-found that the picric acid solution shows the presence of albumen in a specimen diluted considerably beyond the point at which the nitric acid fails to give any indication. The picric acid, too, often causes an immediate albuminous opalescence in specimens in which nitric acid only slowly, and after an interval of some minutes, gives a similar, but sometimes a doubtful, indication." Dr. Johnson also describes a new process by which picric acid can be employed for detecting the presence of glucose in the urine. —*British Medical Journal.*

